# Macrophage Inhibitory Cytokine 1 (MIC-1/GDF15) Decreases Food Intake, Body Weight and Improves Glucose Tolerance in Mice on Normal & Obesogenic Diets

**DOI:** 10.1371/journal.pone.0034868

**Published:** 2012-04-13

**Authors:** Laurence Macia, Vicky Wang-Wei Tsai, Amy D. Nguyen, Heiko Johnen, Tamara Kuffner, Yan-Chuan Shi, Shu Lin, Herbert Herzog, David A. Brown, Samuel N. Breit, Amanda Sainsbury

**Affiliations:** 1 Neuroscience Program, Garvan Institute of Medical Research, Darlinghurst, Sydney, New South Wales, Australia; 2 Department of Immunology, Monash University, Clayton, Victoria, Australia; 3 St Vincent's Centre for Applied Medical Research, St Vincent's Hospital and University of New South Wales, Sydney, New South Wales, Australia; 4 Faculty of Medicine, University of New South Wales, Kensington, Sydney, New South Wales, Australia; 5 School of Medical Sciences, University of New South Wales, Kensington, Sydney, New South Wales, Australia; 6 Sydney Medical School, The University of Sydney, Sydney, New South Wales, Australia; State University of Rio de Janeiro, Biomedical Center, Institute of Biology, Brazil

## Abstract

Food intake and body weight are controlled by a variety of central and peripheral factors, but the exact mechanisms behind these processes are still not fully understood. Here we show that that macrophage inhibitory cytokine-1 (MIC-1/GDF15), known to have anorexigenic effects particularly in cancer, provides protection against the development of obesity. Both under a normal chow diet and an obesogenic diet, the transgenic overexpression of MIC-1/GDF15 in mice leads to decreased body weight and fat mass. This lean phenotype was associated with decreased spontaneous but not fasting-induced food intake, on a background of unaltered energy expenditure and reduced physical activity. Importantly, the overexpression of MIC-1/GDF15 improved glucose tolerance, both under normal and high fat-fed conditions. Altogether, this work shows that the molecule MIC-1/GDF15 might be beneficial for the treatment of obesity as well as perturbations in glucose homeostasis.

## Introduction

Macrophage inhibitory cytokine-1 (MIC-1/GDF15), also known as GDF15, PLAB, NAG-1 or PTGFB, is a divergent member of the TGF-beta family that was identified on the basis of increased expression with macrophage activation [1]. *In vivo* and *in vitro* experimentation suggests that MIC-1/GDF15 probably plays an anti-inflammatory role, notably in mouse models of arthritis and atherosclerosis [2]. In humans its circulating levels are increased in chronic inflammatory diseases such as rheumatoid arthritis and atherosclerosis [2]. Indeed, elevated MIC-1/GDF15 levels are an important risk factor for cardiovascular disease, as well as a marker of poor outcomes and sub-optimal responses to therapy [3]. MIC-1/GDF15 is also expressed by many common cancers, and its serum levels rise approximately in proportion to the stage and extent of disease, providing a potential clinical tool to aid in prevention, diagnosis and prognosis [4]. Serum levels of MIC-1/GDF15 are an independent predictor of all cause mortality [5]. Substantial elevation of circulating MIC-1/GDF15 levels in cancers and other diseases such as chronic renal or cardiac failure are associated with a lower body mass index and sometimes cachexia [2], [6], suggesting that apart from any role in inflammation in disease, MIC-1/GDF15 may also play a role in body weight regulation.

Xenograft of MIC-1/GDF15 expressing human prostate cancer cells into mice leads to loss of fat and lean body mass, and this appears to be directly due to decreased food intake [6]. Administration of anti-MIC-1/GDF15 neutralizing antibodies completely reversed the effects of xenograft-derived MIC-1/GDF15, confirming that the effects were directly mediated by MIC-1/GDF15 production. Weight loss and anorexia could also be induced acutely in mice by administration of recombinant MIC-1/GDF15, an effect mediated via the direct action of MIC-1/GDF15 in areas of the brain that regulate appetite [6]. Interestingly, people with anorexia nervosa or obesity also exhibit elevated circulating MIC-1/GDF15 levels, and obese people with type 2 diabetes exhibit still further elevations in MIC-1/GDF15 compared to non-diabetic obese patients [7]. These findings suggest that in addition to the central regulation of food intake, MIC-1/GDF15 may play a role in regulating metabolism and glucose homeostasis.

Besides activated macrophages, MIC-1/GDF15 is also produced by organs and tissues involved in the control of metabolism, notably the liver and white adipose tissue [8]. This further suggests that MIC-1/GDF15 could be a metabolic regulator. In white adipose tissue, both macrophages of the stromal vascular fraction and adipocytes release MIC-1/GDF15, indicating that it also acts as an adipokine. Adipokines such as adiponectin and leptin, both of which regulate MIC-1/GDF15 release from adipocytes [8], are involved in the regulation of body weight and insulin sensitivity [9]. An additional regulator of both MIC-1/GDF15 release and energy homeostasis is insulin. Circulating MIC-1/GDF15 levels were significantly increased after a two-hour euglycemic hyperinsulinemic clamp in normal control and obese subjects, as well as in those with anorexia nervosa [10]. An inverse correlation between circulating MIC-1/GDF15 levels and insulin sensitivity was also observed, with less insulin sensitive subjects having higher circulating MIC-1/GDF15 levels, further suggesting that MIC-1/GDF15 may regulate peripheral metabolism.

While the above-mentioned reports show increased circulating levels of MIC-1/GDF15 under conditions of altered adiposity and insulin responsiveness, whether MIC-1/GDF15 is a cause or a consequence of these metabolic alterations remains unknown. To help clarify this issue we determined the effects of chronically increased MIC-1/GDF15 levels on food intake, body weight, body composition, energy metabolism and glucose homeostasis, both under conditions of a normal chow and an obesogenic (high fat) diet, using mice overexpressing MIC-1/GDF15 under the control of the macrophage-specific colony-stimulating factor-1 receptor promoter (MIC-1^fms^) versus wild type control mice (MIC-1^+/+^).

## Results

### MIC-1/GDF15 overexpression is associated with a lean phenotype and hypophagia

**Figure 1:**
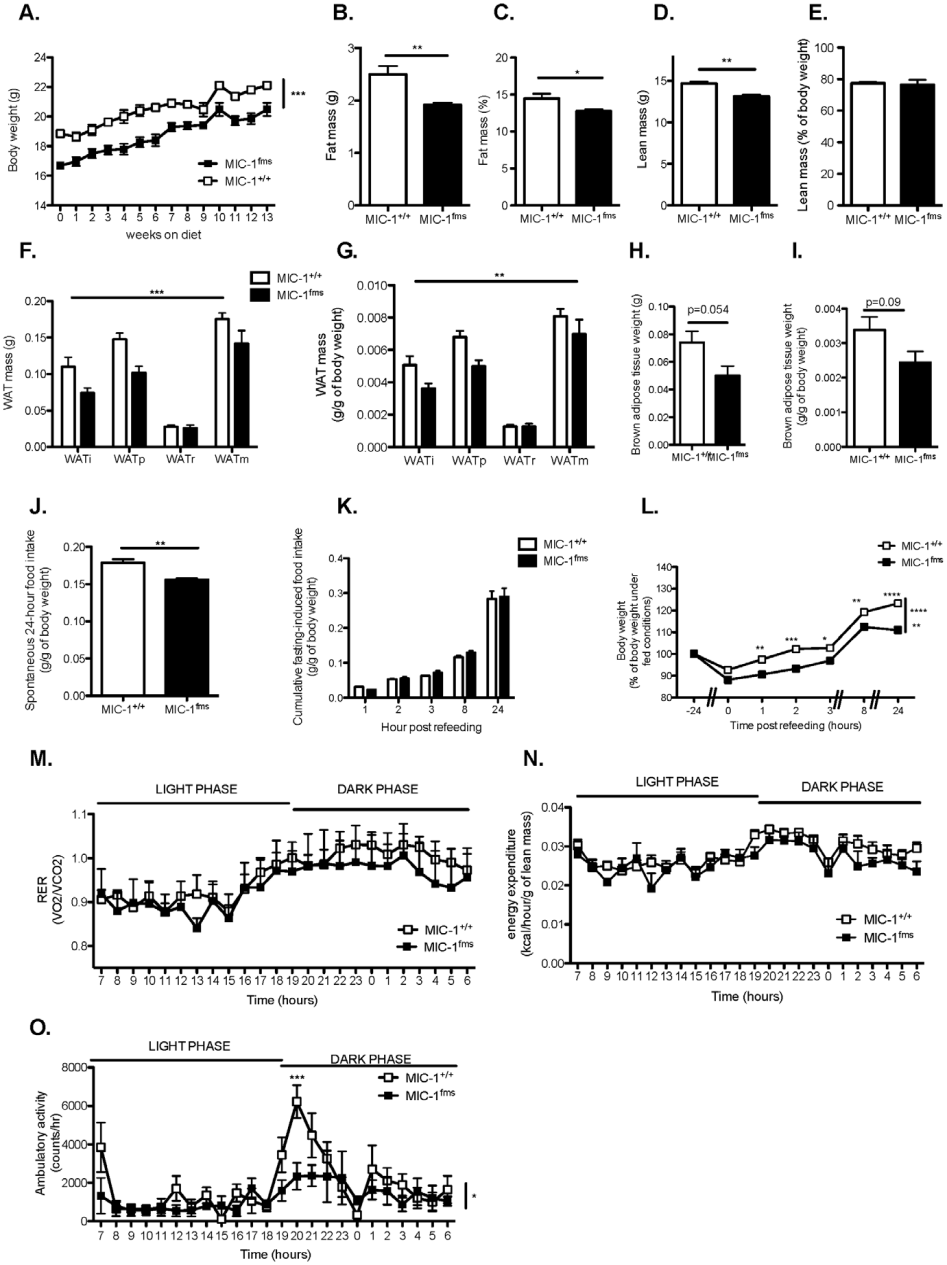
MIC-1/GDF15 overexpression reduces body weight, adiposity and food intake without altering metabolism. A. Body weight of mice overexpressing MIC-1/GDF15 (MIC-1^fms^) and control mice (MIC-1^+/+^) from 11 to 24 weeks of age, represented as 0–13 weeks on the normal chow diet. B–E. Absolute and relative (as a percent of body weight) fat and lean mass as determined by dual energy X-ray absorptiometry (DXA) in normal chow-fed MIC-1^fms^ and MIC-1^+/+^ control mice at 26 weeks of age. F–I Mass of white adipose tissue (WAT) and interscaptular brown adipose tissue depots as absolute weight (F, H) or normalized to body weight (G, I) in normal chow-fed MIC-1^fms^ and MIC-1^+/+^ control mice at 26 weeks of age. i, inguinal; p, periovarian; r, retroperitoneal and m, mesenteric WAT depots. J–K. Spontaneous (J) and cumulative 24-hour fasting-induced food intake (K), normalized to body weight, measured over 24 hours in normal chow-fed MIC-1^fms^ and MIC-1^+/+^ control mice at 25 weeks of age. L. Body weight of 25 week-old normal chow-fed MIC-1^fms^ and MIC-1^+/+^ control mice before 24 hour fasting and at the indicated time points after re-introduction of food, with 100% representing pre-fasting body weight. M–O. Respiratory exchange ratio (RER, M), energy expenditure normalized to lean mass as determined by DXA (N) and ambulatory activity (O) of normal chow-fed MIC-1^fms^ and MIC-1^+/+^ control mice at 26 weeks of age. Data are means ± SEM of 5 female mice per group. *p<0.05, **p<0.01 and ***p<0.001 for the difference between genotypes.

In mice on the normal chow diet, overexpression of MIC-1/GDF15 lead to a significant reduction in body weight from 11 to 24 weeks of age (Fig. 1A). This reduction in body weight in the MIC-1^fms^ transgenic mice was correlated with decreases in absolute (Fig. 1B) and relative (Fig. 1C) whole body fat mass as determined by dual energy X-ray absorptiometry (DXA) at 26 weeks of age. The absolute lean mass of MIC-1/GDF15 transgenic mice was also significantly reduced relative to that of wild type controls (Fig. 1D), but not when normalized to their reduced body weight (Fig. 1E), demonstrating a disproportionate decrease in fat but not lean mass in the transgenic animals. The reduced fat mass of transgenic mice, as determined by DXA, was associated with significant decreases in the mass of dissected white adipose tissue (WAT) depots, both when expressed as absolute weight (Fig. 1F), or when normalized to body weight (Fig. 1G). The absolute (Fig. 1H) and normalized (Fig. 1I) mass of brown adipose tissue (BAT) of MIC-1/GDF15 transgenic mice was not significantly reduced compared to that of control mice.

In order to investigate the reasons for their leaner phenotype, we first looked at food intake in MIC-1^fms^ mice. Indeed, 24-hour spontaneous food intake, either normalized to body weight (Fig. 1J), or expressed as an absolute value (data not shown), was significantly reduced. However, the anorexigenic effect of MIC-1/GDF15 was not seen during re-feeding after a 24-hour fast, either when food intake was expressed as absolute weight (Fig. 1K) or as a percent of body weight (data not shown), suggesting that MIC-1/GDF15 has anorexigenic effects mainly under non-fasted conditions. Interestingly, compared to wild type controls, mice overexpressing MIC-1/GDF15 lost significantly more weight and exhibited significantly delayed weight regain after 24-hour fasting (Fig. 1L). The lean phenotype of the normal chow-fed MIC-1/GDF15 transgenic mice did not appear to result from alteration of their metabolic phenotype, as the respiratory exchange ratio (RER) of transgenic animals was similar to that of control mice (Fig. 1M), indicating similar use of lipids and carbohydrates as energetic fuel sources. Energy expenditure normalized to lean mass was also similar between MIC-1/GDF15 transgenic and control mice (Fig. 1N). Finally, MIC-1/GDF15 transgenic mice exhibited significantly decreased physical activity relative to that of control mice at the start of the dark phase (Fig. 1O), indicating that the lean phenotype of the transgenic mice was not due to hyperactive behaviour. Overall, these results show that transgenic overexpression of MIC-1/GDF15 in normal chow-fed mice is associated with a lean phenotype due to decreased food intake but not to alteration of energy metabolism.

### Overexpression of MIC-1/GDF15 improves glucose tolerance

**Figure 2:**
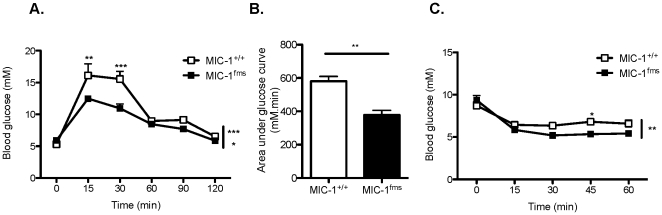
MIC-1/GDF15 overexpression improves glucose tolerance and response to insulin. A. Blood glucose concentrations in response to i.p. glucose injection (1 g/kg) in normal chow-fed mice overexpressing MIC-1/GDF15 (MIC-1^fms^) and control mice (MIC-1^+/+^) at 23 weeks of age. B. Area under the curve calculated from the glucose tolerance test in (A). C. Blood glucose concentrations in response to i.p. insulin injection (1 U/kg) in normal chow-fed MIC-1^fms^ and MIC-1^+/+^ mice at 24 weeks of age. Data are means ± SEM of 5 female mice per group. *p<0.05, **p<0.01 and ***p<0.001 for the difference between genotypes.

Differences in body weight and composition are frequently associated with alterations in glucose tolerance. We thus measured the ability of normal chow-fed MIC-1/GDF15 transgenic mice to clear glucose from the circulation using an intraperitoneal glucose tolerance test. We found a significant improvement in glucose tolerance in the transgenic mice at early time points after glucose injection (Fig. 2A), with the resultant area under the glucose curve being significantly lower in transgenic versus control mice (Fig. 2B). MIC-1/GDF15 transgenic mice also demonstrated significantly reduced blood glucose levels in response to an intraperitoneal insulin tolerance test (Fig. 2C), suggesting that the improved glucose tolerance of these mice may be due to improved insulin responsiveness. We did not observe any significant difference in non-fasted serum insulin levels in normal chow-fed MIC-1^fms^ transgenic versus MIC-1^+/+^ control mice (51.5±10.3 pM in MIC-1^fms^ versus 69.1±19.1 pM in controls, n = 5 mice per group). Weight gain, glucose intolerance and reduced insulin responsiveness are hallmarks of obesity. We thus aimed to determine whether MIC-1/GDF15 transgenic overexpression would have beneficial effects on body weight and glucose homeostasis under obesogenic conditions.

### MIC-1/GDF15 reduces body weight and adiposity under obesogenic conditions

**Figure 3:**
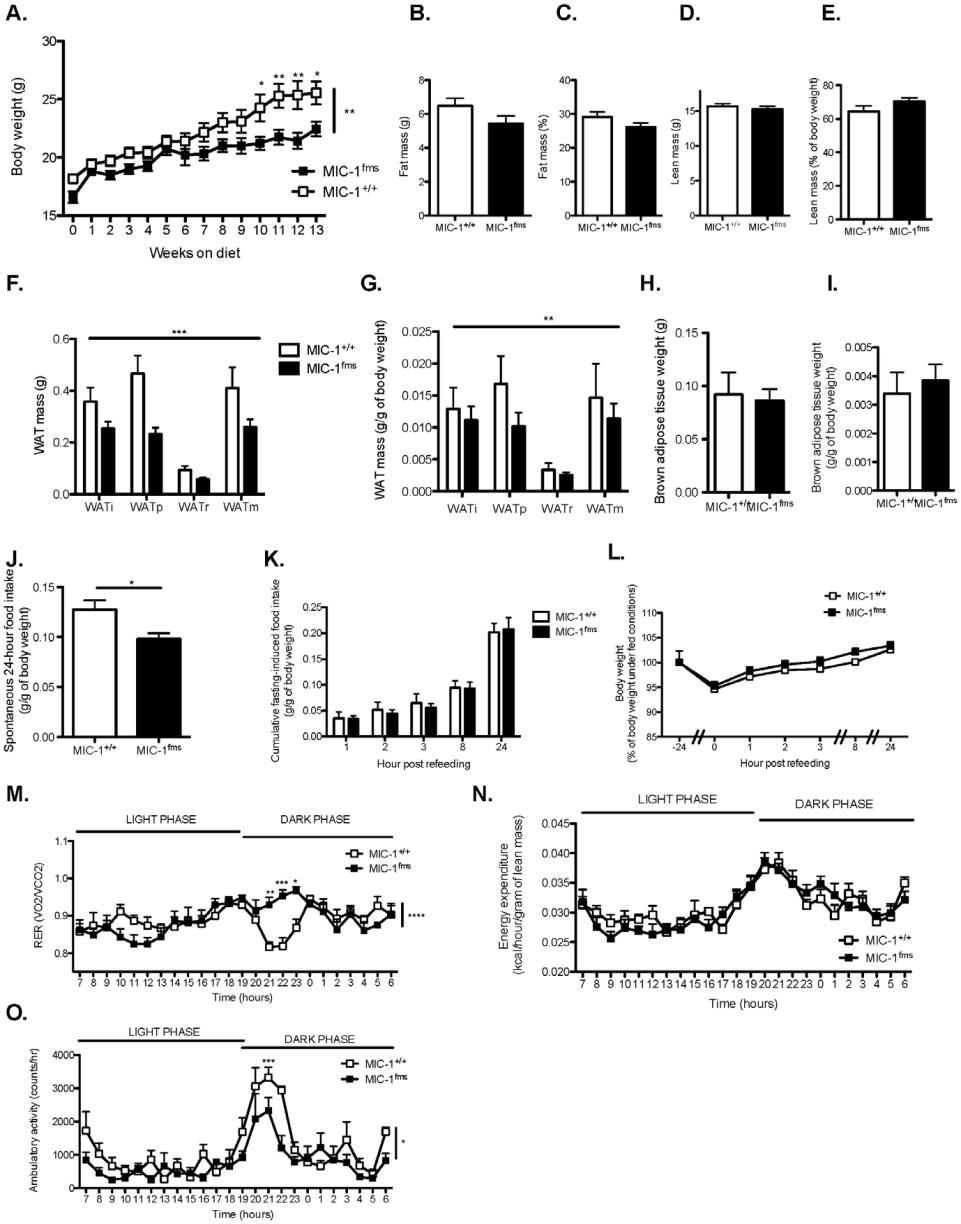
MIC-1/GDF15 overexpression reduces body weight, adiposity and food intake in high fat-fed mice. A. Body weight of mice overexpressing MIC-1/GDF15 (MIC-1^fms^) and control mice (MIC-1^+/+^) from 11 to 24 weeks of age, at 0–13 weeks on a high fat diet. B–E. Absolute and relative (as a percent of body weight) fat and lean mass as determined by dual energy X-ray absorptiometry (DXA) in MIC-1^fms^ and MIC-1^+/+^ control mice at 26 weeks of age, after 15 weeks on the high fat diet. F–I Mass of white adipose tissue (WAT) and interscapular brown adipose tissue depots as absolute weight (F, H) or normalized to body weight (G, I) in high fat-fed MIC-1^fms^ and MIC-1^+/+^ control mice at 26 weeks of age. i, inguinal; p, periovarian; r, retroperitoneal and m, mesenteric WAT depots. J–K. Spontaneous (J) and cumulative 24-hour fasting-induced food intake (K), normalized to body weight, measured over 24 hours in high fat-fed MIC-1^fms^ and MIC-1^+/+^ control mice at 25 weeks of age. L. Body weight of 25 week-old high fat-fed MIC-1^fms^ and MIC-1^+/+^ control mice before 24 hour fasting and at the indicated time points after re-introduction of food, with 100% representing pre-fasting body weight. M–O. Respiratory exchange ratio (RER, M), energy expenditure normalized to lean mass as determined by DXA (N) and ambulatory activity (O) of high fat-fed MIC-1^fms^ and MIC-1^+/+^ control mice at 26 weeks of age. Data are means ± SEM of 5 female mice per group. *p<0.05, **p<0.01 and ***p<0.001 for the difference between genotypes.

Our high fat diet induced significant increases in body weight and adiposity in both MIC-1^+/+^ control mice and MIC-1^fms^ transgenic animals. For instance, body weight and % adiposity (as determined by DXA) at 24–26 weeks of age in chow-fed MIC-1^+/+^, high fat-fed MIC-1^+/+^, chow-fed MIC-1^fms^ and high fat-fed MIC-1^fms^ animals was 22.1±0.14, 25.55±0.98, 20.48±0.44 and 22.44±0.61 g and 14.46±0.65, 29.12±1.46, 12.76±0.24 and 26.1±1.29%, respectively (data are means±SEM of 5 female mice per group. p<0.01 for the effects of genotype, diet and the interaction). It is noteworthy that MIC-1/GDF15 transgenic mice fed a high fat diet retain a significantly lower body weight relative to wildtype counterparts, particularly from the tenth week on the diet onwards (Fig. 3A). Contrary to what was observed in the normal chow fed group, the absolute and relative total body fat mass (Fig. 3B–C) and lean mass (Fig. 3D–E) of high fat-fed MIC-1/GDF15 transgenic mice – as determined by DXA – were not significantly reduced relative to that of control mice. However, the absolute (Fig. 3F) and relative (Fig. 3G) weights of individual dissected WAT depots were significantly reduced in transgenic versus wild type mice at the end of the experiment. In contrast to the WAT, BAT mass was similar between MIC-1/GDF15 overexpressing mice and controls (Fig. 3H–I). As was also observed under conditions of a normal chow diet, MIC-1/GDF15 transgenic mice fed a high fat diet exhibited significantly reduced food intake, either when normalized with body weight (Fig. 3J) or as absolute values (data not shown). Thus, the anorexigenic effect of transgenic MIC-1/GDF15 overexpression is not dependent on the caloric level of the diet. However, this anorexigenic effect depends on the prevailing nutritional status, because after a 24-hour fast, the MIC-1/GDF15 transgenic mice had a similar intake of the high fat diet to that of controls (Fig. 3K), similar to data observed in normal chow-fed animals (Fig. 1K). Contrary to what was observed in normal chow-fed animals, weight loss after fasting was similar between mice overexpressing MIC-1/GDF15 and control mice on the high fat diet (Fig. 3L).

Metabolism of the high fat-fed MIC-1/GDF15 transgenic mice was impaired, as indicated by their RER being significantly different from that of control mice (Fig. 3M). However, energy expenditure normalized to lean mass was similar between genotypes (Fig. 3N). Finally, similar to observations in normal chow-fed animals, the MIC-1/GDF15 transgenic mice on a high fat diet exhibited significantly decreased ambulatory activity, notable during the first part of the dark phase (Fig. 3O). Altogether, these data suggest that MIC-1/GDF15 overexpression leads to a leaner phenotype under obesogenic conditions, probably due to decreased food intake.

### MIC-1/GDF15 overexpression improves glucose tolerance in mice on a high fat diet

**Figure 4:**
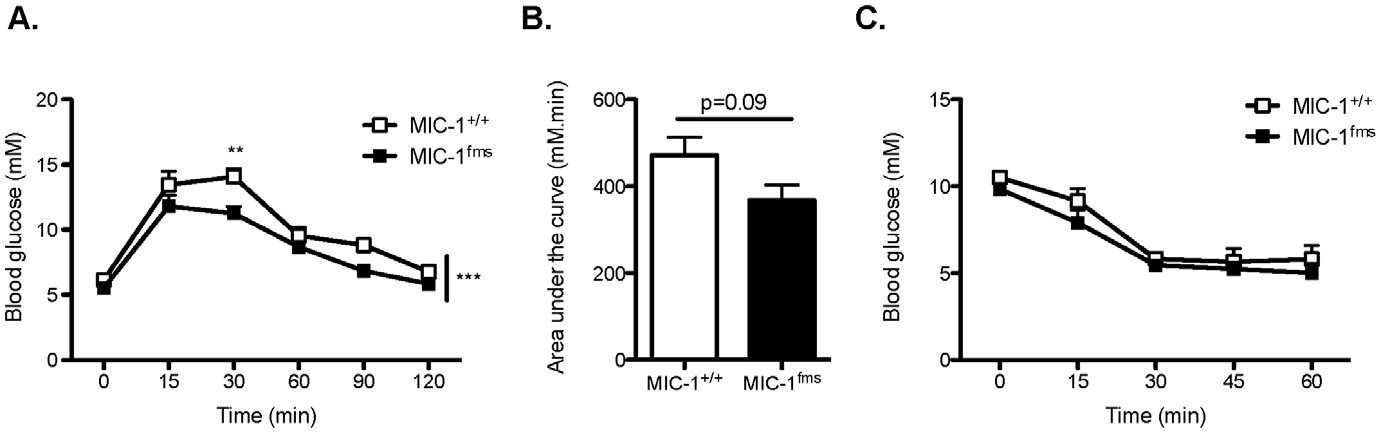
MIC-1/GDF15 overexpression improves glucose tolerance in mice on a high fat diet. A. Blood glucose concentrations in response to i.p. glucose injection (1 g/kg) in mice overexpressing MIC-1/GDF15 (MIC-1^fms^) and control mice (MIC-1^+/+^) at 23 weeks of age, after 13 weeks on a high fat diet. B. Area under the curve calculated from the glucose tolerance test in (A). C. Blood glucose concentrations in response to i.p. insulin injection (1 U/kg) in MIC-1^fms^ and MIC-1^+/+^ mice at 24 weeks of age, after 14 weeks on a high fat diet. Data are means ± SEM of 5 female mice per group. *p<0.05, **p<0.01 and ***p<0.001 for the difference between genotypes.

As we observed under normal chow fed conditions, in high fat-fed mice the overexpression of MIC-1/GDF15 significantly improved glucose tolerance in response to intraperitoneal glucose injection (Fig. 4A). The area under the curve of the glucose tolerance test was decreased in the high fat-fed MIC-1/GDF15 transgenic mice compared to corresponding control mice, but this fell just short of statistical significance (Fig. 4B). Unlike in chow-fed animals, this improvement in glucose tolerance was not likely due to increased insulin responsiveness, as the change in blood glucose during an insulin tolerance test was not significantly different between genotypes (Fig. 4C). As in the normal chow-fed animals, we did not observe any significant difference in non-fasted serum insulin levels in MIC-1^fms^ transgenic versus MIC-1^+/+^ control mice on the high fat diet (62.7±9.0 pM in MIC-1^fms^ versus 115.3±24.6 pM in controls, n = 5 mice per group, p = 0.07). Altogether these data show that the overexpression of MIC-1/GDF15 improves glucose tolerance, both under chow-fed conditions as well as under obesogenic conditions.

## Discussion

In the present study we demonstrate that long-term elevated expression of MIC-1/GDF15 in mice leads to decreases in food intake, body weight and adiposity with concomitantly improved glucose tolerance, both under normal and obesogenic dietary conditions. As these mice do not exhibit any increases in energy expenditure or ambulatory activity, the lean phenotype of mice overexpressing MIC-1/GDF15 likely results from the anorexigenic effect of MIC-1. These results suggest a promising therapeutic potential for MIC-1/GDF15 in the treatment of obesity and perhaps in pre-diabetic glucose intolerance.

Unlike other members of the TGF-beta superfamily, which have been shown to modulate body weight and composition by directly influencing adipose tissue development and function, our data suggest that MIC-1/GDF15 mediates its effects by decreasing food intake. For instance, mice that are deficient in SMAD4, the canonical TGF-beta signalling pathway molecule that is used by most TGF superfamily members, do not exhibit hypophagia. Instead, their reduced body weight is likely due to alterations in white and brown adipose tissue metabolism [11]. We could find no evidence that MIC-1/GDF15 has peripheral effects on adipose tissue metabolism. The respiratory exchange ratio of MIC-1/GDF15 transgenic animals was not decreased, as would have been expected if their lean phenotype were mediated by greater fat oxidation [12]. Bone morphogenic protein-7 (BMP-7), another member of the TGF-beta superfamily, has been shown to mediate weight loss by promoting brown adipose tissue (BAT) development. Indeed, mice with increased BMP-7 expression had higher BAT mass contributing to the associated increase in energy expenditure [13]. We observed no such effect in MIC-1/GDF15 transgenic mice, which exhibited either relatively decreased or unchanged brown adipose tissue mass and similar energy expenditure compared to syngenic controls, both under the normal or obesogenic diets. Taken together, these results suggest that overexpression of MIC-1/GDF15 may not contribute to leanness due to peripheral effects of MIC-1/GDF15 on white or brown adipose tissue development or functionality.

This work shows that like other TGF-beta family members, MIC-1/GDF15 might be a promising target to reduce body weight under obese conditions with a major anorexigenic effect. It is interesting to note that contrary to the anorexigenic cytokine leptin, to which peripheral resistance develops from 8 weeks on a high fat diet [14], there is no obvious resistance to the anorexigenic effects of MIC-1/GDF15 even after 14 weeks on the high fat diet, when MIC-1/GDF15 transgenic mice still eat less than congenic controls. We have previously shown that the anorexigenic effects of MIC-1/GDF15 are mediated through a direct effect on hypothalamic arcuate nucleus neurons by a 47% increase in the expression of pro-opiomelanocortin (POMC), the precursor to the anorexigenic alpha melanocyte stimulating hormone (α-MSH), and a 34% decrease in that of the orexigenic neuropeptide Y (NPY), and that this process involves binding to TGF-beta receptor II [6]. The current work extends these findings by showing that this effect of MIC-1/GDF15 on POMC and NPY expression might be overwhelmed in fasted conditions, where hypothalamic arcuate nucleus POMC expression is reduced and that of NPY is upregulated [15], because the MIC-1/GDF15 transgenic mice do not exhibit reduced food intake after fasting. Moreover, if MIC-1/GDF15 has a stronger effect on POMC than on NPY neurons, as indicated by the changes in POMC and NPY expression in the arcuate nucleus as described above [6], then increased POMC expression may be a major contributor to the phenotype of MIC-1^fms^ mice, as POMC knockout animals exhibit an obese phenotype [16] whereas NPY knockouts remains lean under basal conditions on a normal chow fed [17]. Thus, long-term MIC-1/GDF15 overexpression has sustained anorexigenic effects under both normal and obesogenic conditions, but these effects are not observed in conditions of re-feeding after fasting.

Beneficial roles of MIC-1/GDF15 overexpression are not restricted to reduced body weight and adiposity, as we also show improved glucose tolerance in MIC-1/GDF15 transgenic mice. This effect of MIC-1/GDF15 overexpression is more likely due to improved insulin action rather than increased insulin secretion, because the hypoglycaemic response to insulin was enhanced in MIC-1/GDF15 transgenic animals, at least under normal chow-fed conditions, and because transgenic mice showed no evidence of increased circulating insulin levels. Lean mass and fat mass have been shown to modulate glucose homeostasis, with greater lean mass or reduced fat mass being associated with improved glucose tolerance. Both under normal and obesogenic conditions, MIC-1/GDF15 overexpressing mice have a similar percentage lean mass compared to control mice, demonstrating that MIC-1/GDF15 does not improve glucose tolerance by modulating lean mass. In contrast, the possible contribution of reduced adiposity to the improved glucose tolerance of MIC-1/GDF15 transgenic mice cannot be excluded. Additionally, the effect of MIC-1/GDF15 on glucose homeostasis could be mediated via central mechanisms as described for insulin [18], as is the case for its effects on food intake. Further work would be required to test this hypothesis. It is of interest that the effects of MIC-1/GDF15 over expression on glucose and insulin tolerance were more pronounced in animals on the chow diet than on the high fat diet. The effects of MIC-1/GDF15 to increase hypothalamic POMC expression and decrease that of NPY [6] could conceivably contribute to the improved glucose tolerance or heightened response to insulin in MIC-1^fms^ mice. Indeed, administration of agents that mimic the action of alpha melanocyte stimulating hormone (α-MSH), the anorexigeneic product of the POMC gene, improves the response to insulin in rats [19], whereas central administration of NPY to rats induces muscle insulin resistance [20]. However, because chronic consumption of a high fat diet significantly influences hypothalamic POMC and NPY expression in rodents [21], [22], such changes could contribute to attenuation of the effects of MIC-1/GDF15 over expression on glucose homeostasis under high fat feeding conditions. Taken together, we show that MIC-1/GDF15 improves glucose tolerance by a mechanism likely to involve improved insulin action rather than increased secretion, and that this effect may be mediated by reduced adiposity as well as by a possible role of the central nervous system.

Altogether, this study shows that long-term overexpression of MIC-1/GDF15 reduces body weight and adiposity and improves glucose homeostasis under normal and obesogenic conditions. Thus, MIC-1/GDF15 might provide the basis for a promising therapeutic to improve obesity and its associated metabolic complications.

## Materials and Methods

### Ethics statement and animals

All research and animal care procedures were approved by the Garvan Institute/St Vincent's Hospital Animal Experimentation Ethics Committee (Ethics No: HH #08/01) and were in agreement with the Australian Code of Practice for the Care and Use of Animals for Scientific Purpose. Methods for generation of the MIC-1/GDF15 overexpressing mice on a C57BL6J background were published previously [6]. Overexpression of MIC-1 was under the control of the macrophage-specific colony-stimulating factor-1 receptor promoter (fms), and hence transgenic mice are referred to as MIC-1^fms^. C57BL/6J mice (ARC, Canning Vale, WA, Australia) were used as controls, and these are referred to as MIC-1^+/+^. We have previously shown that compared to MIC-1^+/+^ control mice, MIC-1^fms^ have an over 35-fold increase in MIC-1 mRNA levels in the spleen, and an approximately 90-fold increase in relative serum MIC-1 levels, a fold increase that has been observed in patients with cancer [6]. Mice were housed under conditions of controlled temperature (22°C) and illumination (12-h light cycle, lights on at 0700 h). Unless otherwise stated, mice had *ad libitum* access to food and water. The diet was either normal chow (6% calories from fat, 21% calories from protein, 71% calories from carbohydrates, 2.6 kcal/kg; Gordon's Specialty Stock Feeds, Yanderra, NSW, Australia) or a high fat diet (43% calories from fat, 17% calories from protein, 40% calories from carbohydrate, 4.7% calories from crude fibre, 4.7% calories from acid detergent fibre, 4.78 kcal/kg; Specialty Feeds, Glen Forrest, WA, Australia). The high fat diet was commenced at 10 weeks of age. All experiments were performed on female mice.

### Assessment of body weight and composition

Mice were weighed once a week from the age of 11 weeks to 24 weeks. Upon completion of indirect calorimetry and physical activity measurements as described below, animals were anesthetized with isoflurane and scanned using dual-energy X-ray absorptiometry (DXA) (Lunar PIXImus; GE Healthcare, WI, USA) to determine whole body fat and lean mass. The head was excluded from analyses of body composition. Animals were 26 weeks of age at the time of DXA analysis. Three days following DXA, mice were killed by cervical dislocation and decapitation, and the left inguinal, left periovarian and left retroperitoneal white adipose tissue (WAT) depots, as well as the whole mesenteric WAT and the whole interscapular brown adipose tissue (BAT) depot were removed and weighed. Data are expressed as absolute weight or as grams per gram of body weight.

### Measurement of spontaneous and fasting-induced food intake

At 25 weeks of age, mice were transferred to litter-free individual cages in order to accurately determine actual food intake independently of the amount of food spilled on the cage floor. Spontaneous 24-hour food intake measurements represent an average of 3 days of measuring the amount of food taken from the hopper minus the amount of food spilled. Fasting-induced feeding was measured after fasting the mice for 24 h. Actual food intake was measured as for spontaneous food intake at 1, 2, 3, 8 and 24 hours after reintroduction of food, and is expressed as cumulative food intake. Body weight was measured at each time point before and after fasting.

### Indirect calorimetry

Metabolic rate was measured by indirect calorimetry using an eight-chamber open-circuit calorimeter (Oxymax Series; Columbus Instruments, Columbus, OH, USA). Pre-weighed mice at 26 weeks of age were housed individually in specially built Plexiglass cages (20.1×10.1×12.7 cm). Temperature was maintained at 22°C with airflow of 0.6 1.min^−1^. Mice were singly housed for food intake measurements before transferring into Plexiglass cages and were acclimatized to the cages for 24 h before recordings commenced. Mice were subsequently monitored in the system for 24 h. Oxygen consumption (VO2) and carbon dioxide production (VCO2) were measured every 27 min. The respiratory exchange ratio (RER) was calculated as the quotient of VCO2/VO2, with 100% carbohydrate oxidation resulting in a value of 1, and 100% fat oxidation resulting in a value of 0.7 [23], [24]. Energy expenditure (kcal heat produced) was calculated as calorific value (CV) x VO2, where CV is 3.815+1.232× RER [25], and the result was normalized to lean mass as determined by DXA (described above). Data for the 24-h monitoring period was averaged for 1-h intervals for RER and energy expenditure.

### Measurement of physical activity

During indirect calorimetry, ambulatory activity was also evaluated within the metabolic chambers using an OPTO-M3 sensor system (Columbus Instruments), whereby ambulatory counts were a record of consecutive adjacent photo-beam breaks. Cumulative ambulatory counts of X and Y directions were recorded every minute and summed for 1-h intervals. The analysis was made on mice of 26 weeks.

### Glucose Tolerance Test

At 23 weeks of age, mice were fasted overnight and glucose (Astra Zeneca, North Ryde, NSW, Australia) was injected intraperitoneally at a dose of 1 g/kg. Blood glucose was measured with the AccuCheck™ blood glucose meter (Roche Diagnostics, Mannheim, Germany) using blood samples taken from the tip of the tail at the indicated time points.

### Insulin Tolerance Test

At 24 weeks of age, mice were fasted for at least 5 hours (9:00 to 2.00–4:00 hours) and insulin (Novo Nordisk Pharmaceuticals, Baulkham Hills, Australia) was injected intraperitoneally at a dose of 1 U/kg. Blood glucose concentrations were determined as described above using tail blood samples taken at the indicated time points.

### Statistical Analyses

Data were analyzed with t-tests or 2-way ANOVA followed by Bonferroni post hoc tests if the genotype or interaction effect was significant. Statistical analyses were performed with Prism (GraphPad Software, Inc, LaJolla, USA). Differences were regarded as statistically significant if p<0.05.
